# Case of a 6-year-old boy with anaphylaxis induced by erythritol with positive skin prick test and negative basophil activation test

**DOI:** 10.1186/s13223-022-00670-6

**Published:** 2022-03-24

**Authors:** Satomi Mori, Kazuyuki Kurihara, Chisato Inuo

**Affiliations:** 1 Department of General Medicine, Kanagawa Children’s Medical Center, 2-138-4, Mutsukawa, Minami-ku, Yokohama, Kanagawa 232-8555 Japan; 2 Department of Allergy, Kanagawa Children’s Medical Center, Yokohama, Kanagawa Japan

**Keywords:** Erythritol, Basophil activation test, Skin prick test, Allergy

## Abstract

Erythritol is widely used as an additive in foods and pharmaceuticals. We present the case of a 6-year-old boy who developed an allergy to erythritol. He showed a positive skin prick test result and a negative basophil activation test result. In cases involving allergens with low molecular weights, the test results should be carefully interpreted.

To the Editor,

Erythritol, a type of sugar alcohol, is widely used as an additive in the food and pharmaceuticals because its sweetness is 70% as that of sucrose, and it is calorie-free (0.2 kcal/g). The molecular weight of erythritol is only 122.12 Da. A substance with a simple structure and low molecular weight is typically not recognized by immunoglobulin E (IgE) antibodies. Therefore, it is less likely to induce allergy symptoms. There have been several case reports of allergic reactions induced by erythritol [1–5]. Erythritol allergy was first reported by Hino et al. [1] in 2000. The mechanism by which erythritol causes an allergic reaction remains unclear. Some reports suggested that the basophil activation test (BAT) was useful in evaluating allergic symptoms caused by substances with low molecular weight, such as drugs [6]. Previous reports of erythritol allergy showed that the basophils in these cases reacted to erythritol [2–4]. We present the case of a patient with a positive skin prick test (SPT) result and a negative BAT result for erythritol.

A 6-year-old boy who experienced three anaphylactic episodes of an unknown cause was referred to our department. He had mild bronchial asthma, atopic dermatitis, and allergic rhinitis. He developed eyelid edema and wheezing after consuming a large amount of chewing gum. After consuming a low-calorie jelly drink twice, he experienced eyelid edema, vomiting, wheezing, coughing, and urticaria. During the third episode, he was administered an intramuscular epinephrine injection for anaphylaxis.

An allergic reaction to erythritol was suspected because the foods he consumed contained erythritol. The SPT conducted using the jelly product that the patient had consumed (erythritol concentration: 35 mg/mL), revealed a negative result. Furthermore, SPTs were performed using five concentrations of erythritol solution dissolved in 0.9% saline (1, 10, 100, 200, and 300 mg/mL). Only the 300 mg/mL erythritol solution induced a wheal with a diameter of 4.5 mm. The wheal diameters of the positive control (10 mg/mL histamine dihydrochloride) and negative control (0.9% saline) were 4 mm and 0 mm, respectively. Two healthy subjects without allergies were unreactive to all samples.

In the present case, the BAT was performed with the Allergenicity Kit (Beckman Coulter, Fullerton, CA, USA) according to the method described in a previous report [3]. A whole blood sample was incubated with serially diluted concentrations of erythritol for 1 h. The expression of CD203c on CD3−/CD294+ basophils was analyzed using fluorescence-activated cell sorting (Fig. 1). The CD203c-expresssing cells of the patient’s basophils were not affected by erythritol. The CD203c-expressing cells comprised 3.2%, 2.2%, and 2.1% of the solutions with erythritol concentrations of 5, 0.5, and 0.05 mg/mL, respectively (positive control, 28.6%). An oral food challenge was performed using erythritol in a starting doubling dose of 250 mg every 30 min. The patient developed eyelid edema, lip swelling, and cough with wheezing 20 min after ingesting 1 g of erythritol. The patient was diagnosed with an allergy to erythritol based on the results of the oral food challenge. He showed a positive SPT result and a negative BAT result.

**Fig. 1:**
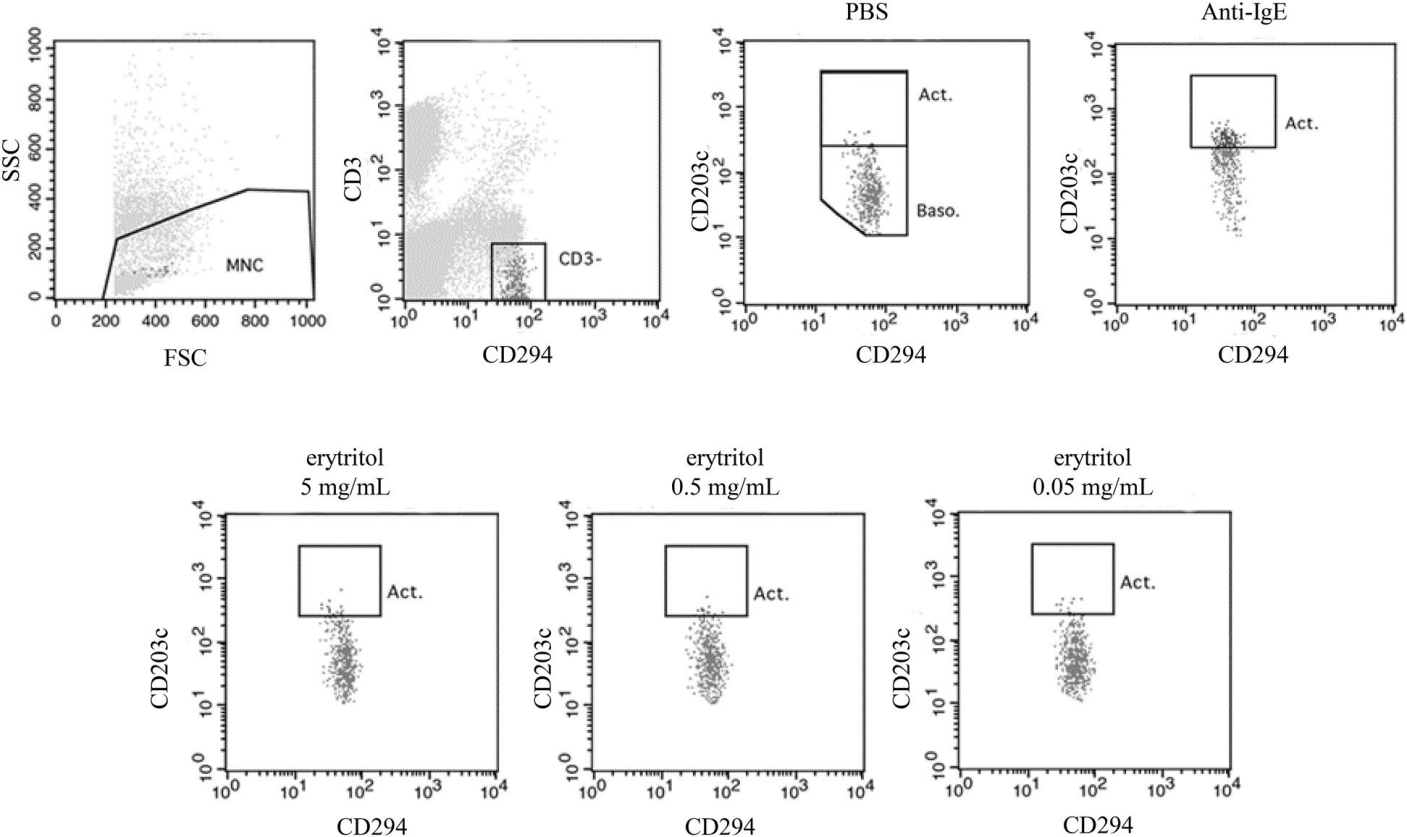
Results of the basophil activation test of the patient. A whole blood sample of the patient was incubated with serially diluted concentrations of erythritol for 1 h. The expression of CD203c on CD3−/CD294+ basophils was analyzed using fluorescence-activated cell sorting. Among the patient’s basophils, the CD203c-expressing cells were not affected by erythritol. The CD203c-expressing cells comprised 3.2%, 2.2%, and 2.1% of the solutions with erythritol concentrations of 5, 0.5, and 0.05 mg/mL, respectively (positive control, 28.6%)

The BAT has been reported to be widely used for the diagnosis of allergies [7]. However, it is important to interpret the results of basophil activation (BA) involving low-molecular-weight compounds, such as drugs and food additives, carefully. Since BA depends on the type of drug or drug metabolite, the BA-positive criteria for drugs is lower than that for food and inhaled allergens [8]. Some reports demonstrated that BA was positive against erythritol in patients with erythritol allergy [2–4]. However, one report showed that the expression of CD203c was low (approximately 11%) at the highest erythritol concentration. Some reports showed that patients with severe drug-induced anaphylaxis showed positive SPT results but were unresponsive to the BAT [9]. Previous reports have had that BATs were less sensitive than skin tests for assessing hypersensitivity to antibiotics, which are low-molecular-weight products similar to erythritol [10]. Thus, the BAT is less sensitive to drug allergies than protein allergens, such as inhaled and food allergens [6]. A negative BAT result cannot exclude an allergy to a low-molecular-weight allergen, such as erythritol.

A previous report showed that BA was suppressed by wortmannin in patients with erythritol hypersensitivity, suggesting the contribution of IgE-mediated pathway to that hypersensitivities [11]. The present patient showed a negative BAT result, suggesting that there might be also a non-IgE-mediated pathway that induces erythritol hypersensitivity.

Few reports have documented adverse reactions to erythritol, and the most reported cases involved Japanese patients [2–4]. Traditional Japanese food products such as soy sauce, miso bean paste, and rice wine contain erythritol. Moreover, erythritol was first approved as a food additive in Japan [12]. Therefore, Japanese individuals would be more likely exposed to higher amounts of erythritol for longer periods, and thus, were more sensitized to erythritol [13]. Currently, erythritol is approved and marketed in more than 60 countries. Furthermore, Japanese cuisine, especially traditional Japanese food, is becoming popular. Anaphylaxis induced by erythritol has also been recently reported for the first time in Europe [5]. Furthermore, Urban et al. [14] suggested that adverse clinical incidents, such as stevia-induced adverse events, are more often related to erythritol hypersensitivity.

This study had some limitations. First, we did not perform a double-blind oral food challenge test. However, the present case experienced several allergic episodes only after ingesting foods that contained erythritol. Second, the highest concentration of erythritol used for the BAT was 5 mg/mL. Although higher concentrations might have elicited a reaction, previous reports documented reactions toward the concentrations included in the present case [4].

It is essential to investigate the mechanism of erythritol allergy to prevent it comprehensively. Erythritol should be considered as a possible cause of food allergy. Moreover, the results of the in vitro test, SPT, and BAT for erythritol should be interpreted carefully. In the present case, the patient showed a positive SPT result but a negative BAT result. The results of this study can contribute to the understanding of the mechanism of erythritol allergy.

## Data Availability

The data used and analyzed during the current study are available from the corresponding author on reasonable request.

## Abbreviations

BA: Basophil activation
BAT: Basophil activation test
IgE: Immunoglobulin E
SPT: Skin prick test
Da: Dalton
